# On the need for tuning the dosimetric leaf gap for stereotactic treatment plans in the Eclipse treatment planning system

**DOI:** 10.1002/acm2.12656

**Published:** 2019-06-21

**Authors:** Laure Vieillevigne, Catherine Khamphan, Jordi Saez, Victor Hernandez

**Affiliations:** ^1^ Department of Medical Physics Institut Claudius Regaud Institut Universitaire du Cancer de Toulouse Toulouse France; ^2^ Centre de Recherches et de Cancérologie de Toulouse UMR1037 INSERM ‐ Université Toulouse 3 – ERL5294 CNRS Oncopole Toulouse France; ^3^ Medical Physics Unit Institut Sainte Catherine Avignon France; ^4^ Department of Radiation Oncology, Hospital Clınic de Barcelona Barcelona Spain; ^5^ Department of Medical Physics Hospital Sant Joan de Reus IISPV Tarragona Spain

**Keywords:** dosimetric leaf gap, dynamic conformal arc, stereotactic treatments, tongue‐and‐groove modeling, volumetric modulated arc therapy

## Abstract

The dosimetric leaf gap (DLG) and tongue‐and‐groove (T&G) effects are critical aspects in the modeling of multileaf collimators (MLC) in the treatment planning system (TPS). In this study, we investigated the dosimetric impact of limitations associated with the T&G modeling in stereotactic plans and its relationship with the need for tuning the DLG in the Eclipse TPS. Measurements were carried out using Varian TrueBeam STx systems from two different institutions. Test fields presenting MLC patterns with several MLC gap sizes (meanGap) and different amounts of T&G effect (TGi) were first evaluated. Secondly, dynamic conformal arc (DCA) and volumetric modulated arc therapy (VMAT) deliveries of stereotactic cases were analyzed in terms of meanGap and TGi. Two DLG values were used in the TPS: the measured DLG (DLG_meas_) and an optimal DLG (DLG_opt_). Measured and calculated doses were compared according to dose differences and gamma passing rates (GPR) with strict local gamma criteria of 2%/2 mm. The discrepancies were analyzed for DLG_meas_ and DLG_opt_, and their relationships with both TGi and meanGap were investigated. DCA arcs involved significantly lower TGi and larger meanGap than VMAT arcs (*P* < 0.0001). By using DLG_meas_ in the TPS, the dose discrepancies increased as TGi increased and meanGap decreased for both test fields and clinical plans. Dose discrepancies dramatically increased with the ratio TGi/meanGap. Adjusting the DLG value was then required to achieve acceptable calculations and configuring the TPS with DLG_opt_ led to an excellent agreement with median GPRs (2%/2 mm) *>* 99% for both institutions. We also showed that DLG_opt_ could be obtained from the results of the test fields. We demonstrated that the need for tuning the DLG is due to the limitations of the T&G modeling in the Eclipse TPS. A set of sweeping gap tests modified to incorporate T&G effects can be used to determine the optimal DLG value.

## 1 INTRODUCTION

Stereotactic body radiation therapy (SBRT) and stereotactic radiosurgery (SRS) treatments are particularly valuable modalities for treating relatively small lesions with high delivered doses. Stereotactic treatments generally use different delivery techniques: the most popular are dynamic conformal arc (DCA) and volumetric modulated arc therapy (VMAT). Some SBRT protocols, such as RTOG 0236^1^ and 0813^2^ required a minimum field size, encouraging multiple static beams or DCA. This requirement may be difficult to fulfill with VMAT as multileaf collimator (MLC) apertures do not strictly follow the projection of the planning target volume (PTV). Thus, VMAT arcs may lead to small MLC gaps. Nevertheless, the use of VMAT in SRS and SBRT is becoming increasingly widespread.^3^ Since the target volumes are typically small, so are the radiation field sizes involved. This can be challenging for the accuracy of the treatment planning system (TPS) calculations.^4^ Hence, the ICRU 91 report^5^ recently recommended rigorous testing of the TPS dose calculation accuracy in stereotactic treatments because lesions can be in proximity to vital sensitive structures.

Dose calculation accuracy is known to be affected by inappropriate handling of simplifications in the TPS algorithms and models.^6^, ^7^, ^8^ For rounded leaf‐end MLC systems, the Eclipse TPS requests the user to input two MLC configuration parameters: the MLC transmission ratio and the dosimetric leaf gap (DLG). Some studies^9^ have found good agreement between calculated and delivered doses by using the DLG measured with sweeping gap tests^10^, ^11^ or the dynamic chair test.^12^ However, other authors^13^, ^14^, ^15^ found substantial discrepancies and reported on the need for tuning the DLG value configured in the Eclipse TPS. Kielar et al.^13^ observed discrepancies between calculated and measured doses around 5% for the Varian's high‐definition multileaf collimator (HDMLC), which were greatly reduced by increasing the DLG entered into the TPS by more than 1 mm. Another important characteristic that can affect dose calculation accuracy is the tongue‐and‐groove (T&G) modeling. Indeed, many MLC models have a T&G design, where the sides of adjacent leaves interlock in order to reduce interleaf transmission. However, this configuration produces underdosage between adjacent leaf pairs in asynchronous MLC movements due to the additional shielding by the tongue of opposing leaf sides during treatment delivery.^16^ This underdosage is known as the T&G effect and it can significantly change the dose distribution.^17^ In arc treatments, T&G effects are typically smoothed out due to the gantry rotation, but they can produce a reduction in average doses of up to 5%–7%.^18^ In a recent study^19^ we used a set of test fields that demonstrated inadequate modeling of the T&G in the Eclipse TPS, remarkably for the HDMLC and small dynamic MLC gaps. SRS or SBRT VMAT arcs may lead to highly irregular MLC patterns and small MLC apertures with individual leaves repeatedly extending into the radiation field and thus presenting high T&G effects. Therefore, careful attention should be given to the T&G modeling in order to reduce dose uncertainties.

The purpose of this study is twofold: (a) to evaluate the impact of limitations associated with the T&G modeling in stereotactic clinical plans and (b) to investigate the relationship between these limitations and the need for tuning the DLG in the Eclipse TPS. In addition, a method to determine the value of the DLG that maximized the agreement between calculations and measurements in stereotactic clinical cases is presented.

## 2 MATERIALS AND METHODS

Measurements and calculations were carried out in two different institutions (A and B). In both institutions, a Varian TrueBeam STx equipped with the HDMLC for a 6 MV WFF photon beam energy with flattening filter (WFF) and at a dose rate of 600 monitor units (MU)/min was used for dose delivery. Flattening filter‐free (FFF) photon beams were also used in one institution for comparison purposes. Doses were calculated using the anisotropic analytical algorithm (AAA) algorithm with a 1 mm calculation grid size in the Eclipse TPS v13 (Varian Medical Systems, USA). The effective spot size parameter was set to 0 mm. An angular resolution of 2 degrees was selected for dose calculations, as used in clinical practice.

### 2.A MLC model in Eclipse

Only two parameters of the MLC model in Eclipse are user configurable, namely the DLG and the MLC transmission. The TPS uses a single value for MLC transmission, which is the average radiation transmitted through the leaves. Regarding the leaf tip, Eclipse accounts for the increased transmission through the leaf tip by applying a shift to the leaf‐end position which amounts to half the DLG value introduced during configuration. Therefore, doses are calculated with an effective gap larger than the nominal gap by a distance equal to the DLG. The procedure recommended by the vendor^11^ to determine the DLG is the sweeping gap test as initially introduced by LoSasso et al.^10^ For that purpose, the vendor supplies DICOM files implementing the tests that can be readily imported into the TPS. Concerning the modeling of the T&G, Eclipse extends the leaf projections in the direction perpendicular to the leaf motion by a certain tongue width, which is subtracted from the delivered fluence. Thus, the field size in the direction of leaf movements is enlarged by the DLG, while in the perpendicular direction it is reduced due to the tongue width by 0.625 mm.^11^, ^20^ This last value is fixed and unmodifiable by the user.

In this study, two DLG values were assessed: the “measured DLG” (DLG_meas_) and the “optimal DLG” (DLG_opt_). DLG_meas_ was obtained with the standard sweeping gap test. In contrast, both institutions determined DLG_opt_ during the SRS/SBRT commissioning process and was defined as the value producing the best agreement between calculations and measurements for a set of stereotactic clinical plans. To that aim, a procedure was followed in which the DLG parameter was increased iteratively until optimal QA results were achieved according to the stereotactic QA program of each institution.

### 2.B Test fields including tongue‐and‐groove effects

The original sweeping gap test involves uniformly extended leaves for different MLC gaps without exposing any of the leaf sides and consequently without any T&G effect. In a previous work,^19^ we designed a set of test fields based on the sweeping gap test that incorporated well‐defined amounts of T&G by applying different shifts to the adjacent leaves. These test fields were: (a) the asynchronous sweeping gap (aSG) for sliding window beams and (b) the asynchronous oscillating sweeping gap (aOSG) for VMAT arcs. The detailed characteristics of these test fields are given in Hernandez et al.^19^ and similar tests have also been proposed by other investigators.^21^, ^22^ For each beam of aSG and aOSG tests, a tongue‐and‐groove index (TGi) was defined as the quotient of the distance between adjacent leaf ends “*s*” and the MLC gap size (meanGap) used: TGi = *s*/meanGap. For a TGi equal to zero, there is no T&G effect and when TGi increases the T&G effect becomes larger. The investigated MLC gap sizes were 10, 20, and 30 mm, which were considered representative of clinical treatments. DICOM plans for the aSG and aOSG tests were created with an in‐house software implemented in MATLAB^®^ (Mathworks, Massachusetts, USA), and imported into the TPS for calculation and delivery. All the tests were calculated with both DLG values with the same number of MUs. Dose measurements were performed with a PTW ion chamber model T31013. This chamber is smaller than the typical Farmer chamber used for measuring the DLG,^23^ but its active length (16 mm) still spanned several leaves, providing an estimate of the average impact of the T&G effect.^19^ The chamber was positioned at the isocenter, 10 cm depth, in a water phantom for the aSG test and in a cylindrical phantom for the aOSG test. Chamber readings were corrected for the daily output variations. The expanded measurement uncertainty *U* 24 was calculated for one standard deviation confidence interval. Measured doses *D*_meas_ were compared to the calculated doses *D*_calc_ in the sensitive volume of the chamber for both DLG settings and dose differences were evaluated as (*D*_calc_ *− D*_meas_)/*D*_meas_. Dose deviations for both tests with respect to meanGap, TGi, and to the ratio TGi/meanGap were investigated. In addition, a DLG value, noted as DLG_minΔD_, was calculated as the value that is required to compensate for the dose discrepancies obtained from test fields for a particular meanGap and TGi.

### 2.C Clinical plans

Five SRS brain and five SBRT lung patients were randomly chosen. The cases included small and large volumes, with PTV volumes ranging from 2 to 40 cc for brain cases (mean volume of 21.5 cc) and from 8.6 to 81 cc for lung cases (mean volume of 26.1 cc). For each patient, a DCA and a VMAT plan were optimized according to our institution's dosimetric clinical guidelines. The two different modalities were selected to highlight potential differences between VMAT and DCA deliveries. Hence, a total of 20 plans (10 DCA and 10 VMAT) were generated. VMAT plans used 4 arcs and DCA plans used between 3 and 5 arcs depending on the complexity of the case. The same plans were used in both institutions and dose calculations were performed with both DLG_meas_ and DLG_opt_ using the same MLC patterns and the same number of MUs.

#### 2.C.1 Dose agreement

Different detectors were used to avoid any source of systematic uncertainties. Thus, measurements were performed with: (a) the 4D Octavius equipped with the 1000 SRS array (PTW, Freiburg, Germany) associated with PTW Verisoft software,^25^ (b) the Delta 4 (ScandiDos, Uppsala), and (c) the Varian portal imager with Varian's portal dose prediction algorithm PDIP (for VMAT plans). Verification plans were generated in Eclipse and gamma passing rates (GPRs) for both DLG settings were compared using paired Student's *t*‐test with statistical significance at *p < *0*.*05. Verisoft software calculates a volumetric gamma (three‐dimensional [3D] gamma) and provides the percentage of points that passes the criteria within the volume defined by a given isodose level. All GPRs were calculated with the 2%/2 mm local gamma criteria with a threshold of 10%. Moreover, the 3D gamma in Verisoft was analyzed for higher thresholds (30%, 80%, and 95%). The standard local and global gamma criteria 3%/3 mm were also recorded although deemed inappropriate for stereotactic treatments, which require stricter criteria. Additionally, qualitative line profiles agreement and the doses at the isocenter were also analyzed. The calculated dose was obtained as the average dose of a volume contoured in the TPS at the sensitive volume of the central 1000 SRS ion chamber. Similarly to the test fields, differences between calculations and measurements were evaluated as (*D*_calc_ *− D_meas_*)/*D*_meas_. Dose distributions were analyzed for each arc and also for the composite plans.

#### 2.C.2 Analysis of clinical plans and relationship with dose agreement

An in‐house software named “Plan‐Analyzer,”^26^ developed in MATLAB^®^, was used to parse the MLC information from the DICOM files. In the present study, the meanGap and the mean TGi were investigated. The meanGap for a given arc was calculated as the average leaf pair opening at each control point weighted by the corresponding fractions of MUs. A similar procedure was followed for TGi, which was defined as the ratio of the difference between adjacent leaf positions and their MLC gap, averaged over all the leaves in the beam and all control points. TGi was set to a maximum value of 1 in case of interdigitation (i.e., for *s > *gap) because the dosimetric impact due to the T&G effect increases linearly with *s* only for *s < *gap.^19^ The relationships between the dose agreement (in terms of GPR and dose differences at the isocenter) with both meanGap and TGi were investigated. To that aim, results were reported with respect to: (a) meanGap, (b) TGi, and (c) TGi/meanGap.

## 3 RESULTS

Using the standard sweeping gap tests, the DLG_meas_ was found to be 0.3 mm (institution A) and 0.4 mm (institution B), which was in agreement with the value obtained with the dynamic chair method. The MLC transmission ratio was 1.25%. Both institutions independently determined an DLG_opt_ of 1.1 mm, each institution using its own set of stereotactic clinical plans. As already mentioned, this DLG_opt_ was obtained during SRS/SBRT commissioning by varying the DLG parameter in the Eclipse TPS iteratively until the best matching was reached between measured and calculated dose distributions for the plans considered.

### 3.A Test fields (aSG and aOSG tests)

#### 3.A.1 Analysis of test fields

**Figure 1:**
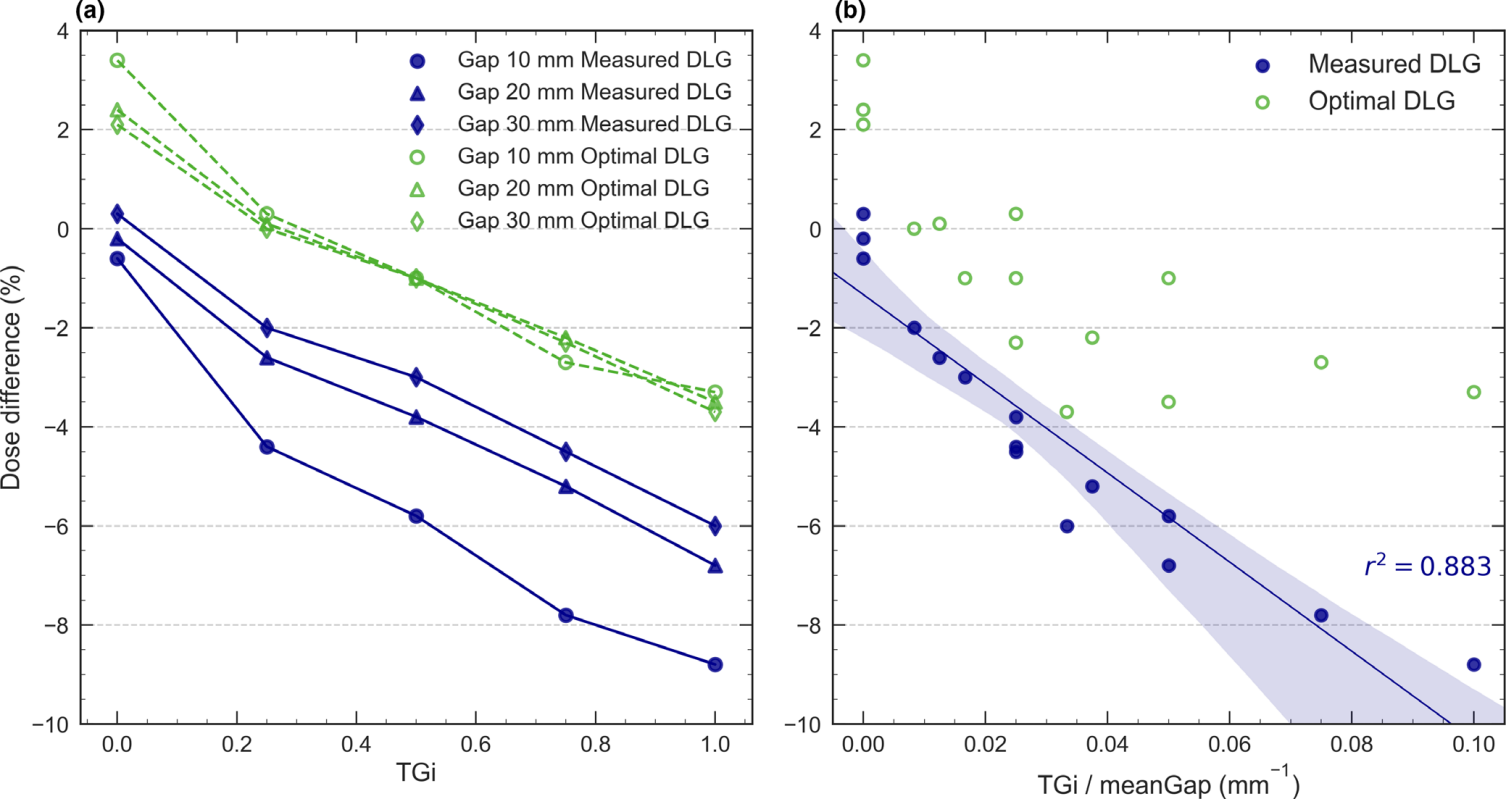
Dose difference between calculations and measurements for the aOSG test with three different MLC gap sizes for 6 MV WFF. Results are shown for both the measured DLG and the optimal DLG as a function of (a) TGi and (b) TGi/meanGap.

Figure 1 illustrates the variation in dose difference for the aOSG test as a function of the TGi and with respect to the ratio TGi/meanGap for both DLG settings. As shown in Fig. 1(a) the dose differences increased as TGi increased and as the MLC gap decreased. In the absence of T&G, a better agreement was found with DLG_meas_. This was expected since the DLG was measured with sweeping gaps without T&G. Nevertheless, as the T&G effect became higher, the discrepancies with DLG_meas_ increased. Some dose differences exceeded 5% and were up to 8% for TGi = 1 and for the smallest gap. In the presence of T&G effects, the agreement clearly improved using DLG_opt_. With a TGi = 0.25, the dose differences were reduced from 2–4% to nearly 0% and with a higher value of TGi = 0.5 they decreased from 3–6% to 1%. It should be noted that the dose differences for DLG_opt_ were less dependent on the gap size than for DLG_meas_. Indeed, the curves related to DLG_opt_ for gaps 10, 20, and 30 mm almost overlapped.

Dose differences clearly depended on the ratio TGi/meanGap for DLG_meas_ [Fig. 1(b)], with a strong linear behavior (*r^2^* = 0*.*883). Discrepancies were larger for the largest ratio, which corresponds to large T&G and small MLC gaps. Tuning the DLG partially compensated for these discrepancies that were noticeably reduced by using DLG_opt_ [see Fig. 1(a)]. Similar results were obtained for the aSG and aOSG tests in both institutions. Both tests were also carried out for the energies 6 MV FFF, 10 MV FFF, and 10 MV WFF obtaining the same behavior as shown in Fig. 1, and are provided as Supporting Information in Fig. S1. The uncertainty *U* on the ion chamber measurement was estimated to be less than 0.5%.

#### 3.A.2 Determination of the DLG_min∆D_

From the results obtained for test fields with DLG_meas_ [Fig. 1(a)], the value DLG_min∆D_ that minimizes dose discrepancies between measurements and calculations can be calculated. Let us consider a dose difference ∆*D* between the measured and calculated doses for an asynchronous sweeping gap field with a particular MLC gap size “gap” and a representative “TGi”:

(1) $$\Delta D=D_{\text{TGi},\text{gap}}^{\text{meas}}-D_{\text{TGi},\text{gap}}^{\text{calc}}$$

This dose discrepancy ∆*D* can be compensated by increasing the DLG parameter by an amount *δ* that can be obtained by linear interpolation with a larger MLC gap (such as 30 mm) as

(2) $$\delta =\frac{\Delta D}{D_{\text{TGi},\text{gap = 30}\;\text{mm}}^{\text{calc}}-D_{\text{TGi},\text{gap}}^{\text{calc}}}\left(\text{30}\;\text{mm}-\text{gap}\right),$$

where $D_{\text{TGi},\text{gap = 30}\;\text{mm}}^{\text{calc}}$ and $D_{\text{TGi},\text{gap}}^{\text{calc}}$ are the calculated doses for a MLC gap of 30 mm and the representative “gap,” respectively, corresponding to a particular “TGi.” The ${\text{DLG}}_{\text{min}\Delta D}$ value that minimizes dose discrepancies can then be easily obtained as

(3) $${\text{DLG}}_{\text{min}\Delta \text{D}}={\text{DLG}}_{\text{meas}}+\delta .$$

Using these expressions for TGi = 0.25, a ${\text{DLG}}_{\text{min}\Delta D}$ of 1.0 mm was obtained for the MLC gap widths of 10 and 20 mm, while for TGi = 0.50, the ${\text{DLG}}_{\text{min}\Delta D}$ was 1.2 mm. These values are in agreement with our iteratively derived ${\text{DLG}}_{\text{opt}}$ of 1.1 mm.

### 3.B Clinical plans

**Figure 2:**
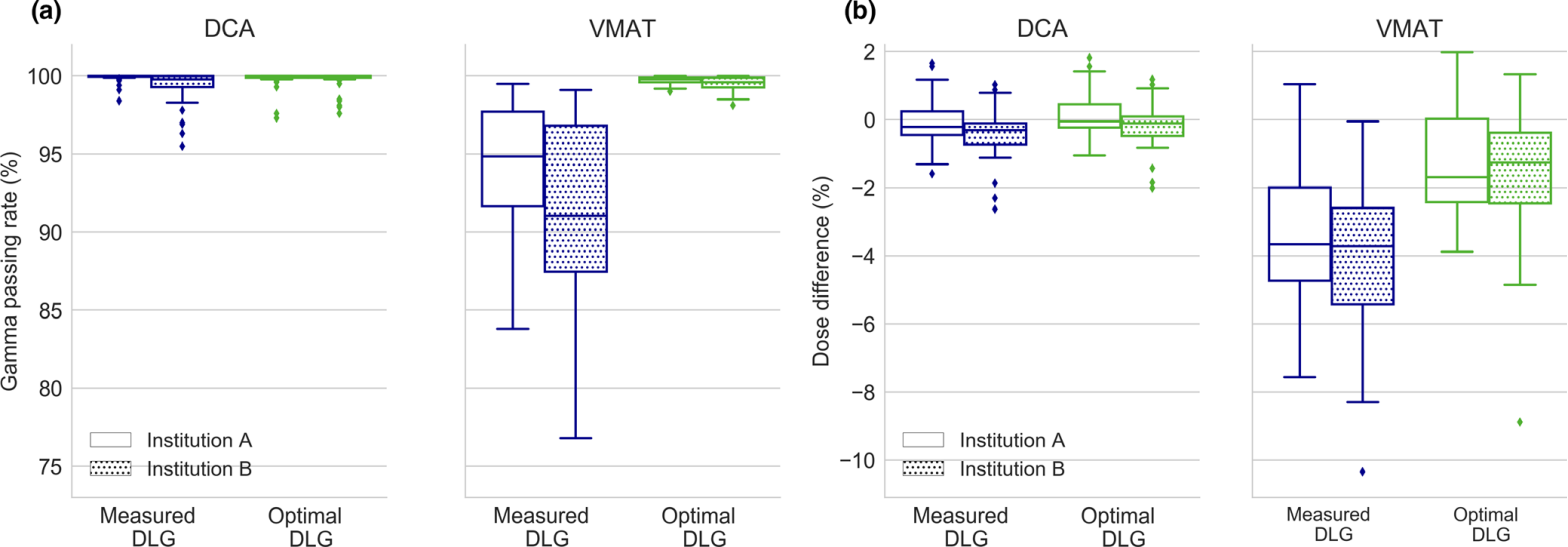
Boxplots for DCA and VMAT arcs of (a) the GPR (2%/2 mm) and of (b) the dose differences at the isocenter obtained with 1000 SRS for both institutions for 6 MV WFF. Central lines indicate the median value, the box limits represent the 1st and the 3rd quartile and the whiskers indicate the minimum and maximum values (outliers are excluded).

All DCA plans exhibited an excellent agreement for both DLG_meas _and DLG_opt_. GPRs (2%/2 mm) were close to 100% for both institutions [Fig. 2(a)] and dose differences were within ± 2% [Fig. 2(b)] regardless of the DLG value used. No significant improvement was found by using DLG_opt_ (*P* = 0*.*23 for 1000 SRS, *P* = 0*.*25 for Delta 4).

**Figure 3:**
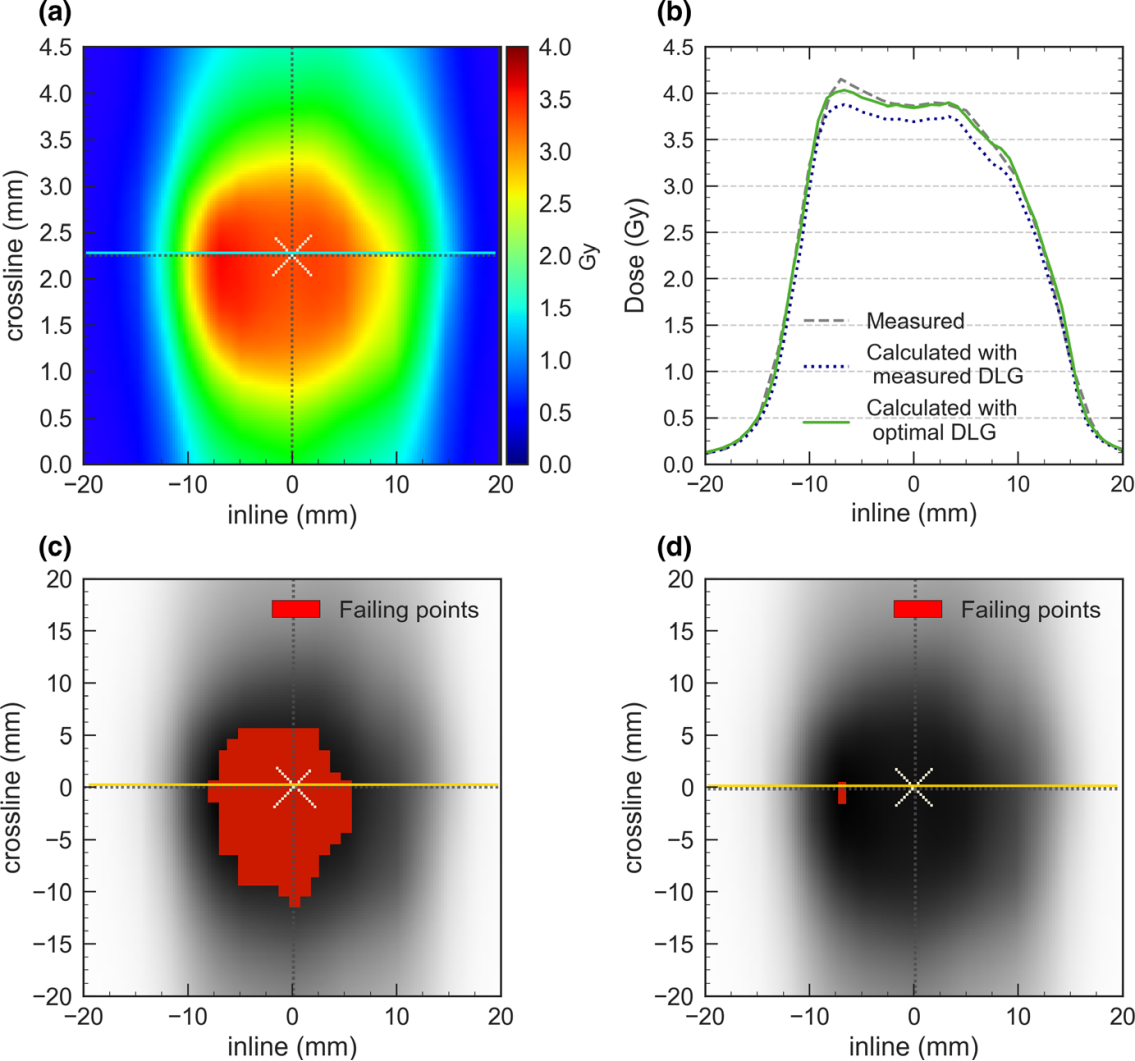
Measurements obtained with 1000 SRS for a representative VMAT treatment for 6 MV WFF: (a) dose distributions in a coronal plane, (b) profiles comparison using the measured and optimal DLG, (c) gamma map using DLG_meas_ and (d) gamma map using DLG_opt_. Pixels failing the gamma criteria (2%/2 mm) are marked and indicate that the measured dose exceeded the planned dose.

For VMAT plans, the GPRs (2%/2 mm) obtained when DLG_opt_ was used in the TPS calculations were higher than those obtained with DLG_meas_. The median GPR increased for both institutions from 90%–95% to over 99% [Fig. 2(a)]. For all detectors, similar results were found and the improvement in GPR with DLG_opt_ was statistically significant with *P* = 0*.*003 with 1000 SRS, *P* = 0*.*005 with Delta 4, and *P* = 0*.*0003 with the portal imager. Results with 1000 SRS improved for all the dose thresholds evaluated (*p < *0*.*003), but the improvement was more significant for the highest thresholds of 80% and 95% (*P* = 0*.*0006 and *P* = 0*.*0001, respectively). Similar results were observed in both institutions with all detectors and for composite arcs. The detailed results from all detectors are provided as Supporting Information in Table S1. DLG_meas_ produced excessively low calculated doses and the difference in the dose at the isocenter was reduced from 4% to 1.5% when DLG_opt_ was used [see Fig. 2(b)]. Figure 3 shows a comparison between a representative dose distribution measured with 1000 SRS and Eclipse calculations. With DLG_meas_, the shape of the dose distribution for VMAT treatment seemed to be adequately reproduced but the TPS calculations underestimated the dose, whereas when DLG_opt_ was used the agreement substantially improved.

#### 3.B.1 Analysis of clinical plans in terms of TGi and meanGap

**Figure 4:**
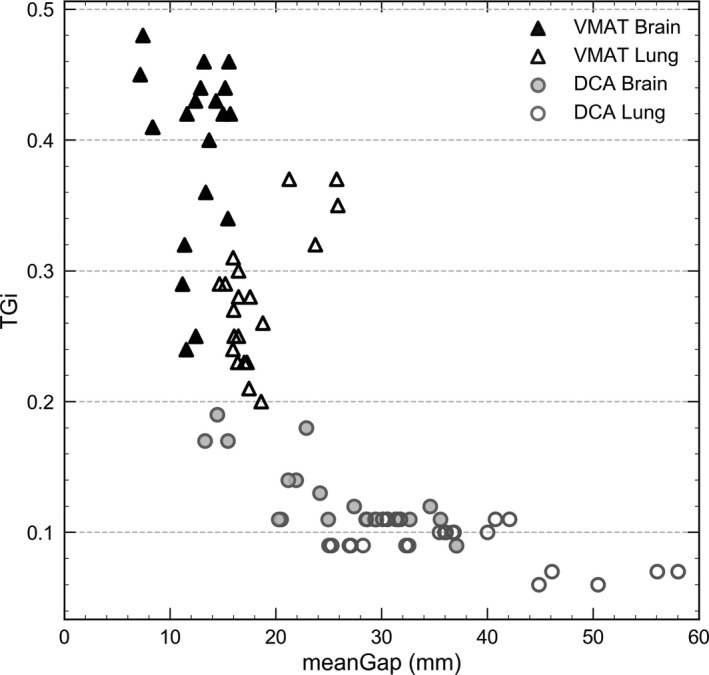
MeanGap and TGi for brain and lung cases for the different techniques and treatment sites.

DCA and VMAT arcs were analyzed in terms of meanGap and TGi and results are shown in Fig. 4 and in Table S2 (provided as Supporting Information). DCA arcs presented TGi less than 0.2 with a median value of 0.11 while VMAT arcs exhibited values ranging between 0.2 and 0.48. For DCA, significantly lower TGi and larger meanGap were found compared to VMAT plans (*p < *0*.*0001). For the largest target volume, a meanGap of around 58 mm was obtained for DCA delivery whereas it remained smaller than 25 mm for VMAT. As it can be seen, a threshold of TGi = 0.2 clearly separates DCA from VMAT arcs. In general, brain plans produced lower meanGap and higher TGi than lung plans. It can be noted that several DCA plans had meanGap ≤ 20 mm, which were very similar to those in VMAT plans, although their TGi was lower.

#### 3.B.2 Relationship between plan analysis and dose agreement

**Figure 5:**
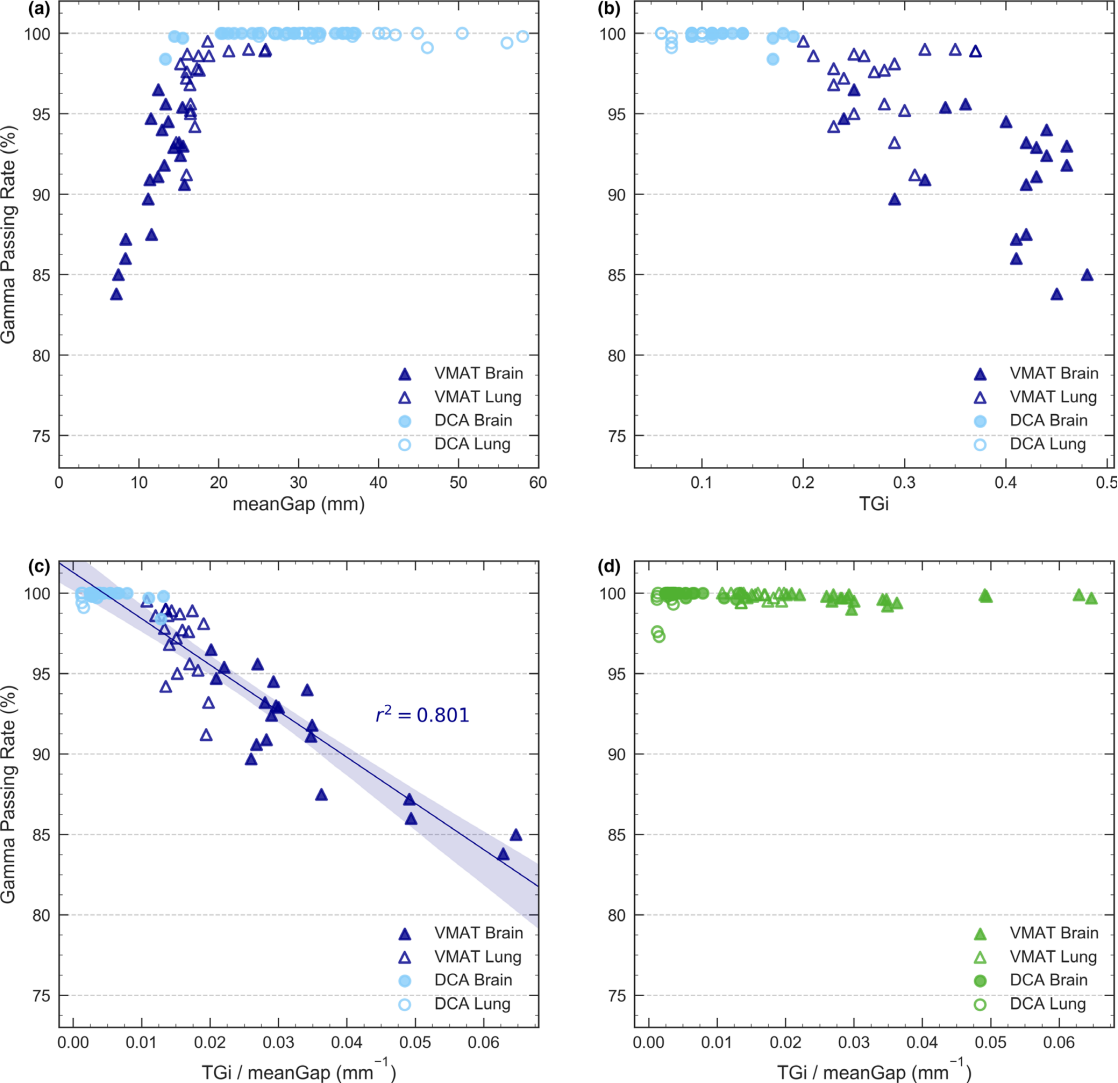
Local GPR for 2%/2 mm obtained with 4D Octavius and 1000 SRS for dynamic confomal arc (DCA) and VMAT arcs for 6 MV WFF. Results obtained with the measured DLG are given as a function of (a) meanGap, (b) TGi and (c) TGi/meanGap. Results with the optimal DLG are shown in (d).

The GPRs (2%/2 mm) obtained for DCA and VMAT arcs with 1000 SRS are given in Fig. 5.

Figure 5(a) clearly shows that for VMAT, the smaller the gap, the lower the GPR. This explains why GPRs for brain cases were lower than for lung cases. However, all results were in good agreement for DCA, even for arcs with meanGap ≤ 20 mm.

Figure 5(b), which presents the GPRs as a function of TGi, clearly separates DCA and VMAT arcs. All results were good for TGi < 0*.*2 (DCA plans), while for TGi > 0*.*2 (VMAT plans) the agreement was worse and the maximum discrepancies increased with TGi. A wide spread in GPRs was found for arcs with similar TGi values, due to differences in their meanGap.

To take into account both TGi and meanGap, the GPRs were represented as a function of the ratio TGi/meanGap [Fig. 5(c)] and showed an almost linear behavior. DCA arcs exhibiting low TGi/meanGap ratios presented good agreement, even for arcs with a low meanGap. For VMAT, in contrast, the drop in GPR was evident as TGi/meanGap increased, similarly to the aSG and aOSG tests. Thereby, for all VMAT arcs, the larger TGi/meanGap ratio, the lower the GPR. In particular, for brain cases, VMAT arcs involved high TGi and small meanGap, producing the highest TGi/meanGap ratios and the lowest GPRs.

As previously commented, an excellent agreement was obtained in all cases with calculations performed with DLG_opt_ [Fig. 5(d)]. All VMAT arcs presented GPR ≥ 98% regardless of their meanGap and TGi, which means that tuning the DLG effectively compensated for those limitations. A few DCA plans yielded slightly less congruous GPRs, but they were still in very good agreement (GPR > 97%).

Similar results were obtained for 6 MV FFF, with slightly better GPRs but exactly the same trends, and are provided as Supporting Information in Fig. S2.

## 4 DISCUSSION

The use of VMAT in SBRT and SRS treatments is rapidly increasing^27^, ^28^, ^29^ as a result of its dosimetric advantages over DCA.^30^, ^31^ However, the VMAT technique poses dosimetric challenges such as small MLC apertures, high T&G effects, and individual leaves repeatedly extending into the radiation field. Subsequently, to ensure accurate dose calculation, careful attention should be given to the MLC modeling and to the DLG value used in the Eclipse TPS configuration.

In this study, we showed that configuring Eclipse with DLG_meas_ may lead to large discrepancies between measurements and calculations in VMAT stereotactic plans. Dose differences were nearly 5% on average for VMAT SRS plans with an underestimation of the TPS dose calculations. These discrepancies might have clinically relevant implications and are unacceptable according to international guidelines and QA protocols.^32^, ^33^, ^34^ It should be noted that good agreement was found for DCA arcs, which present lower T&G effects and larger MLC gaps than VMAT arcs. In DCA, the limited T&G effects were expected since the MLC aperture follows the projection of the PTV with quite uniformly extended leaves. This is not the case for VMAT arcs, where leaves may move back and forth repeatedly thereby increasing T&G effects.

We found that increasing DLG_meas_ in Eclipse by 0.7–0.8 mm greatly reduced discrepancies, producing very good agreement between calculations and measurements (median GPRs > 99%) for VMAT arcs with a stringent local gamma criteria of 2%/2 mm. Eclipse tended to underestimate the calculated dose when DLG_meas_ was used. Increasing the DLG in the TPS increased the calculated doses, consequently improving the agreement between calculations and measurements. This increase in the DLG parameter agrees with findings from other investigators^13^, ^17^, ^35^: Kielar et al.^13^, ^35^ increased the value from 0.5 to 1.7 mm and Kim et al.^13^, ^17^, ^35^ adjusted it from 0.39 to 1.1 mm. Nevertheless, the cause of the discrepancies and the reason to tune the DLG remained unknown to date.

This study investigated the reason why tuning the DLG value was necessary. We demonstrated that dose discrepancies between calculations and measurements were related to limitations in the T&G modeling that could be partially compensated by tuning the DLG. This is, to our knowledge, the first explanation of the need to change the value of the DLG parameter in the Eclipse TPS. Indeed, our results revealed clear relationships between discrepancies and the metrics meanGap and TGi when DLG_meas_ was used. For VMAT, the smaller the meanGap, the larger the dose difference, remarkably for meanGap < 20 mm. In contrast, all DCA plans produced good results even for arcs with meanGap around 15 mm. This can be explained since TGi values for VMAT arcs were in the range 0.2–0.48, while all DCA arcs had TGi < 0*.*2 and the maximum discrepancies increased with TGi and the higher the TGi, the lower the GPR. Tuning the DLG is an effective way to compensate for these discrepancies because they have a similar dependency with the MLC gap size. Thus, the smaller the MLC gaps, the more pronounced the dose underestimation by the Eclipse TPS and the higher the impact of increasing the DLG. Additionally, the small T&G effects produced by DCA are fully located at the periphery of the PTV; this is a high gradient region and, hence, dose differences barely affect the results of QA verifications and are not clinically relevant. Both DLG_meas_ and DLG_opt_ values lead to quite similar dose differences in DCA and thus it appears that adjusting the DLG parameter has no benefit. Only the smallest target sizes present a better agreement between the measured and calculated doses with DLG_opt_. This is probably due to the agreement of small MLC output factors. As demonstrated by Fogliata et al*.* 36, tuning the DLG affects the output factor for small MLC‐defined fields and increasing the DLG improves the calculation accuracy.

The value of the DLG is an important parameter to create a robust model for SRS/SBRT planning and optimizing the DLG value to be introduced in the Eclipse TPS using clinical cases is a tedious and time consuming process. We showed that DLG_opt_ can be obtained by minimizing dose discrepancies in the aSG or the aOSG tests for TGi values representative of clinical plans (0.2–0.5). Thus, apart from measuring the DLG following the vendor's recommended procedure, measurements for a TGi in the range 0.2–0.5 and for gap sizes of 10–20 mm (representatives of clinical treatments) can be carried out and the DLG value that minimizes the obtained differences can be considered as the optimal DLG. The DLG calculated with this procedure was comparable to the value of 1.1 mm obtained from optimizing agreement between measured and calculated dose distributions in clinical plans. Obviously, end‐to‐end tests to validate clinical treatment plans are still required but, in our opinion, this method constitutes a much more straightforward and comprehensive process than optimizing agreement for a set of representative clinical plans.

Nevertheless, tuning the DLG cannot perfectly compensate the limitations in the T&G modeling. Indeed, clinical plans yield different TGi and meanGap parameters and DLG_opt_ should depend on the characteristics of each particular plan. Several investigators already demonstrated that the optimal DLG was plan dependent^14^, ^15^ and our results support and explain their findings. This might help explain why we still found some dose differences around of 2–3% when DLG_opt_ was used. However, these discrepancies might also be due to the presence of dose gradients at the isocenter and to the small active volume of the detectors used. The implications of tuning the DLG in the TPS should be carefully evaluated since it might originate calculation errors in certain cases and affect the MLC field size. For instance, for a sweeping gap of 10 mm without T&G, a 1 mm increase in the DLG will increase the calculated doses by approximately 10%, introducing a 10% discrepancy for that field.^10^ Hence, tuning the DLG may improve the calculation accuracy for a certain range of plan parameters, but it might not work for all plans. This stays an artificial way to compensate for the poor modeling of the T&G effect.

In this study, the DLG parameter was tuned in order to maximize the agreement in SBRT and SRS clinical plans. The DLG_opt_ found worked well for all the stereotactic plans considered but, since the optimal value actually depends on the characteristics of each particular plan, a different DLG_opt_ might be necessary in other cases. Several investigators have indeed proposed the use of a specific MLC configuration for SBRT and SRS different than the configuration used for non SBRT/SRS plans,^13^, ^14^, ^15^ but we recommend that the use of different MLC configurations for different techniques is carefully addressed and investigated in each particular clinical setting.

Measurements were carried out in two institutions and with three QA systems with different characteristics regarding their dosimetric accuracy, active detector volume and spatial resolution, ruling out the possibility that our results are only valid for a particular beam model or due to limitations of a specific QA device. Although composite plans were also evaluated, we presented the analysis of individual arcs because it produced clearer relationships.

This study was conducted with Varian TrueBeam STx systems and was focused on 6 MV WFF photon beams. However, the MLC model implemented in the TPS is the same for all beam energies; therefore, the limitations of the model and the need for tuning are independent of the particular treatment unit and energy used. Results obtained for FFF beams supported this point. The discrepancies will depend on the MLC characteristics and we evaluated the HDMLC because the thinner the leaves, the higher the frequency of T&G effects and the larger the impact of the limitations in the T&G modeling.^19^ This is in agreement with the fact that much lower dose discrepancies have been reported for the Millennium 120 MLC by several investigators.^15^, ^21^

A limitation of the present study is that only the Eclipse TPS with the AAA algorithm was evaluated. However, many TPSs use T&G models similar to Eclipse, where a constant T&G width is added to the lateral leaf edges.^37^, ^38^ We recently showed that for rounded MLCs a model with a variable T&G width (or alternatively, a variable transmission) at the leaf tip was needed to produce accurate calculations.^39^ Therefore, careful attention must be paid to the MLC configuration parameters in plans involving small MLC apertures and high T&G effects. This can also explain why AAPM Practice Guidelines recommend tuning of the MLC configuration parameters to optimize agreement in clinical cases.^40^ The strategy followed in the method proposed in Section 3.A.2 can be applied to other TPSs, but different expressions will be needed depending on the user‐definable parameters used to describe the rounded leaf end in each TPS. Regarding AAA, we focused on this algorithm because in both institutions the Acuros XB (AXB) algorithm was not yet available for clinical use. Both AAA and AXB use the same MLC modeling and thus should suffer from the same limitations regarding the T&G effect and we already showed that they both produced the same differences in the aSG and aOSG tests.^19^ Despite that, a better behavior in clinical cases with AXB might be found, because, as presented by Fogliata et al.^36^ AXB calculations of small MLC‐defined beams output are in better agreement with measurements than AAA and this agreement improves if the DLG is increased.

## 5 CONCLUSIONS

Accurate modeling of the MLC by the TPS is essential, and one of the important aspects is the modeling of the T&G. Calculations for dynamic conformal arcs in SRS and SBRT treatments with the Eclipse TPS are accurate and barely sensitive to the DLG used (DLG_meas_ and DLG_opt_) in the TPS configuration. On the contrary, for VMAT Eclipse tended to underestimate the dose and large dose discrepancies were found when DLG_meas_ was used, making it necessary to tune the DLG parameter in order to achieve clinically acceptable calculations.

A clear relation was found between the amount of T&G in the treatment plans and the dose discrepancies when DLG_meas_ was used. This indicates that the need for tuning the DLG could be due to limitations in the MLC model in the Eclipse TPS, in particular in the modeling of the T&G effect. The aSG and the aOSG tests can be used not only to characterize these limitations,^19^ but also to derive the optimal DLG by minimizing dose differences for clinically representative TGi values. This is a more efficient and pragmatic method than optimizing the agreement for a set of representative clinical plans.

Tuning the DLG is an effective method to compensate for the poor modeling of the T&G and it yielded very good results in all pretreatment verifications. However, the tuning performed may not be valid for all plan characteristics and the implications of tuning this parameter should be carefully validated for each TPS version and dose calculation algorithm. A better T&G model in the Eclipse TPS is needed, especially for the HDMLC and for treatment plans involving irregular apertures and small MLC gaps. This would both increase the accuracy of dose calculations and avoid the need for tuning the DLG, facilitating an easier and more comprehensive configuration of the Eclipse TPS.

## CONFLICTS OF INTEREST

The authors have no relevant conflicts of interest to disclose.

## Supporting information


**Fig. S1. **Dose difference between calculations and measurements for the aOSG test with three different MLC gap sizes for the energies 6 MV FFF, 10 MV WFF, and 10 MV FFF. Results are shown for both the measured DLG and the optimal DLG as a function of (a) TGi and (b) TGi/meanGap.

**Fig. S2.** Local GPR for 2%/2 mm obtained with 4D Octavius and 1000 SRS for DCA and VMAT arcs for 6 MV FFF. Results obtained with the measured DLG are given as a function of (a) meanGap, (b) TGi, and (c) TGi/meanGap. Results with the optimal DLG are shown in (d).

**Table S1. **Average gamma passing rates over arcs for VMAT plans for 6 MV WFF with 4D Octavius and 1000 SRS, portal imagers PDIP and Delta4 related to measured (Meas) and optimal (Opt) DLG values for different gamma criteria (2%/2 mm and 3%/3 mm) with local and global dose normalization.

**Table S2. **MeanGap (mm) and TGi as a function of the technique: DCA and VMAT.
