# Supplementary figures and images for: On the need for tuning the dosimetric leaf gap for stereotactic treatment plans in the Eclipse treatment planning system
Source: J Appl Clin Med Phys. 2019 Jun 21;20(7):68–77. doi: 10.1002/acm2.12656 (PMC6612699; doi:10.1002/acm2.12656)

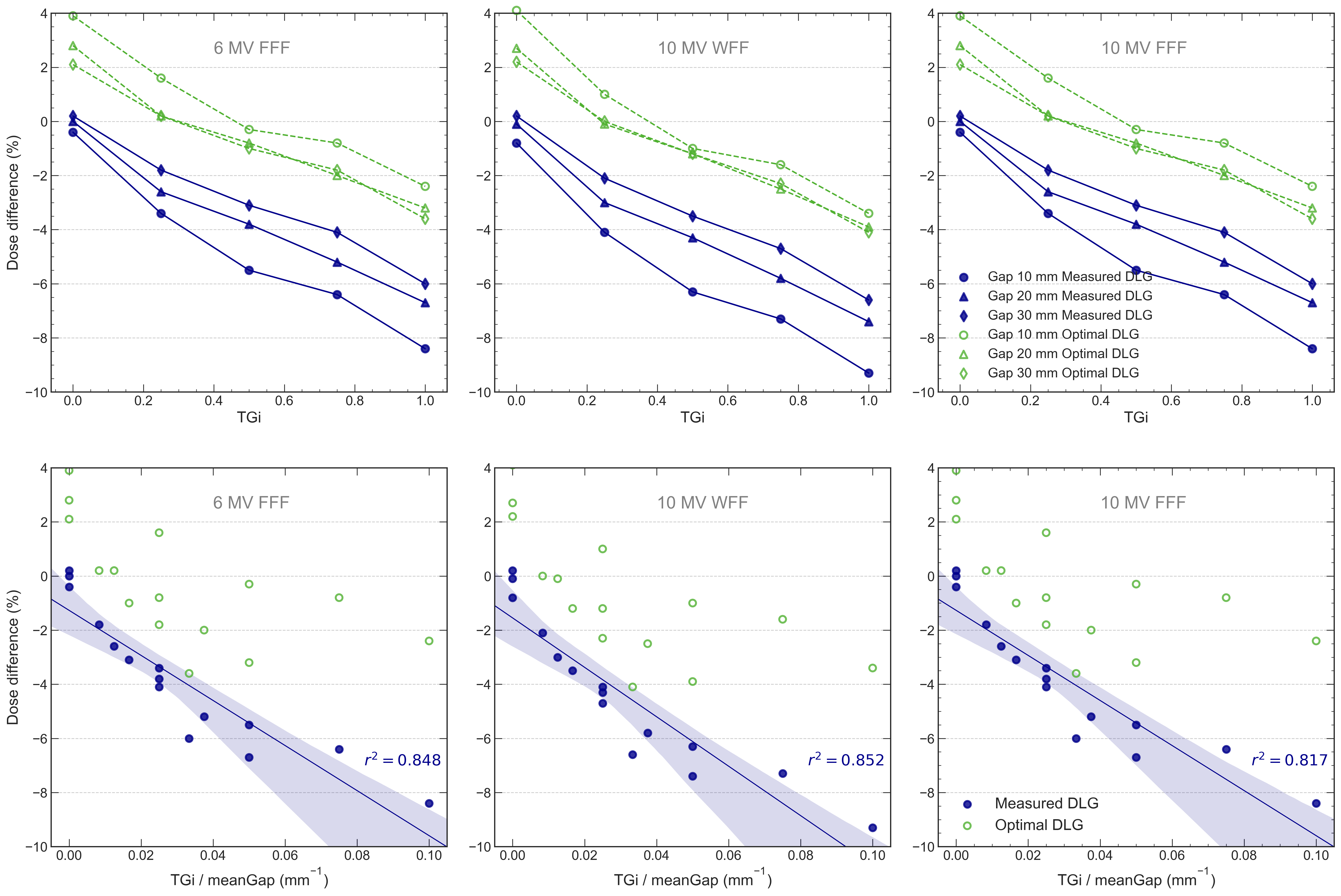

Supplement: Supplementary file 1 — Fig. S1. Dose difference between calculations and measurements for the aOSG test with three different MLC gap sizes for the energies 6 MV FFF, 10 MV WFF, and 10 MV FFF. Results are shown for both the measured DLG and the optimal DLG as a function of (a) TGi and (b) TGi/meanGap. [file ACM2-20-68-s001.png]

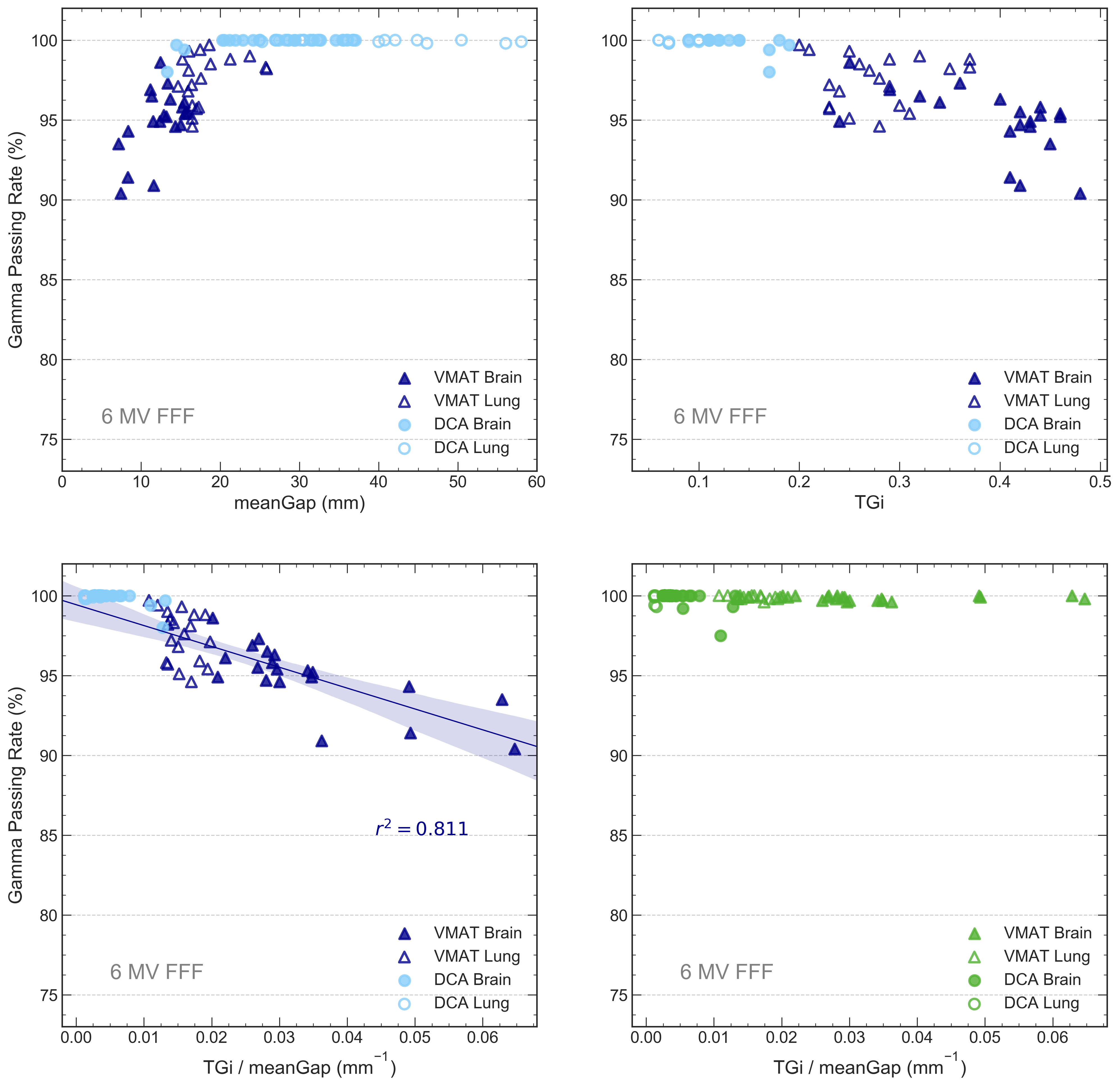

Supplement: Supplementary file 2 — Fig. S2. Local GPR for 2%/2 mm obtained with 4D Octavius and 1000 SRS for DCA and VMAT arcs for 6 MV FFF. Results obtained with the measured DLG are given as a function of (a) meanGap, (b) TGi, and (c) TGi/meanGap. Results with the optimal DLG are shown in (d). [file ACM2-20-68-s002.png]
