# Supplementary material for: On the need for tuning the dosimetric leaf gap for stereotactic treatment plans in the Eclipse treatment planning system
Source: J Appl Clin Med Phys. 2019 Jun 21;20(7):68–77. doi: 10.1002/acm2.12656 (PMC6612699; doi:10.1002/acm2.12656)
Supplement: Supplementary file 3 — Table S1. Average gamma passing rates over arcs for VMAT plans for 6 MV WFF with 4D Octavius and 1000 SRS, portal imagers PDIP and Delta4 related to measured (Meas) and optimal (Opt) DLG values for different gamma criteria (2%/2 mm and 3%/3 mm) with local and global dose normalization. [file ACM2-20-68-s003.docx]

|  | DLG | 4D Octavius and 1000 SRS | | | | PDIP | | | | Delta 4 | | | | |
| --- | --- | --- | --- | --- | --- | --- | --- | --- | --- | --- | --- | --- | --- | --- |
|  |  | 2%/2mm  local | 2%/2mm  global | 3%/3mm  local | 3%/3mm  global | 2%/2mm  local | 2%/2mm  global | 3%/3mm  local | 3%/3mm  global | 2%/2mm  local | 2%/2mm  global | 3%/3mm  local | 3%/3mm  global |  |
| Brain clinical cases | Meas  Opt | 89.2  99.2 | 96.4  99.8 | 97.9  99.9 | 99.4  99.9 | 91.5  98.0 | 92.5  98.6 | 96.2  99.5 | 96.8  99.7 | 94.3  99.8 | 97.6  100 | 98.8  100 | 99.8  100 |  |
|  | Meas  Opt | 86.9  99.4 | 96.5  99.7 | 97.3  100 | 99.2  100 | 94.7  98.7 | 95.2  98.9 | 97.7  99.5 | 97.7  99.5 | 93.0  99.8 | 96.1  100 | 98.9  100 | 99.7  100 |  |
|  | Meas  Opt | 86.2  99.6 | 98.6  99.7 | 97.9  99.9 | 99.6  99.9 | 96.3  99.5 | 95.9  99.3 | 98.3  99.7 | 97.9  99.6 | 99.4  98.6 | 99.7  100 | 100  100 | 100  100 |  |
|  | Meas  Opt | 76.4  99.2 | 95.1  99.8 | 93.2  100 | 99.3  100 | 93.2  98.1 | 93.1  98.0 | 95.9  99.5 | 95.6  99.4 | 96.6  100 | 97.8  100 | 99.7  100 | 99.7  100 |  |
|  | Meas  Opt | 88.6  99.2 | 96.5  99.6 | 99.699.9 | 99.3  99.9 | 90.7  97.5 | 91.6  97.9 | 95.6  99.3 | 96.2  99.3 | 93.9  99.8 | 96.7  100 | 98.9  100 | 77.5  100 |  |
|  |  |  |  |  |  |  |  |  |  |  |  |  |  |  |
| Lung clinical cases | Meas  Opt | 94.4  99.9 | 99.0  99.9 | 99.5  100 | 99.8  100 | 96.6  100 | 96.4  100 | 98.9  100 | 98.8  100 | 99.1  100 | 99.7  100 | 100  100 | 100  100 |  |
|  | Meas  Opt | 97.6  99.9 | 99.6  100 | 99.8  100 | 100  100 | 98.7  99.8 | 98.9  100 | 99.7  100 | 99.8  100 | 96.3  99.4 | 99.2  99.8 | 100  100 | 100  100 |  |
|  | Meas  Opt | 96.4  99.7 | 99  100 | 99.6  100 | 99.9  100 | 91.6  96.1 | 93.5  97.4 | 96.3  98.4 | 97.3  99.1 | 97.4  99.7 | 99.2  100 | 99.7  100 | 100  100 |  |
|  | Meas  Opt | 95.0  98.9 | 97.6  99.0 | 98.7  99.2 | 99.0  99.2 | 99.2  99.7 | 99.1  99.8 | 99.9  100 | 99.9  100 | 97.2  99.8 | 99.6  100 | 99.8  100 | 100  100 |  |
|  | Meas  Opt | 98.8  100 | 99.7  100 | 99.9  100 | 99.9  100 | 93.8  99.3 | 94.2  99.4 | 98  99.8 | 98.1  99.8 | 99.4  99.8 | 99.6  100 | 99.9  100 | 99.9  100 |  |
