# Supplementary material for: On the need for tuning the dosimetric leaf gap for stereotactic treatment plans in the Eclipse treatment planning system
Source: J Appl Clin Med Phys. 2019 Jun 21;20(7):68–77. doi: 10.1002/acm2.12656 (PMC6612699; doi:10.1002/acm2.12656)
Supplement: Supplementary file 4 — Table S2. MeanGap (mm) and TGi as a function of the technique: DCA and VMAT. [file ACM2-20-68-s004.docx]

|  | DCA (47 arcs) | VMAT (40 arcs) |  |
| --- | --- | --- | --- |
| MeanGap (mm) [range] | 30.6 [13.3 – 58] | 15.5 [7.2 – 25.9] | p<0.0001 |
| TGi [range] | 0.11 [0.06 – 0.19] | 0.32 [0.20 – 0.48] | p<0.0001 |
